# Electron Transfer Coupled to Conformational Dynamics in Cell Respiration

**DOI:** 10.3389/fmolb.2021.711436

**Published:** 2021-08-06

**Authors:** Marco Reidelbach, Christoph Zimmer, Brigitte Meunier, Peter R. Rich, Vivek Sharma

**Affiliations:** ^1^ Department of Physics, University of Helsinki, Helsinki, Finland; ^2^ Department of Structural and Molecular Biology, University College London, London, United Kingdom; ^3^ Institute for Integrative Biology of the Cell (I2BC), Université Paris-Saclay, Gif-sur-Yvette, France; ^4^ HiLIFE Institute of Biotechnology, University of Helsinki, Helsinki, Finland

**Keywords:** proton pumping, molecular dynamics simulations, density functional theory, yeast bioenergetics, mitochondrial respiration

## Abstract

Cellular respiration is a fundamental process required for energy production in many organisms. The terminal electron transfer complex in mitochondrial and many bacterial respiratory chains is cytochrome *c* oxidase (C*c*O). This converts the energy released in the cytochrome *c*/oxygen redox reaction into a transmembrane proton electrochemical gradient that is used subsequently to power ATP synthesis. Despite detailed knowledge of electron and proton transfer paths, a central question remains as to whether the coupling between electron and proton transfer in mammalian mitochondrial forms of C*c*O is mechanistically equivalent to its bacterial counterparts. Here, we focus on the conserved span between H376 and G384 of transmembrane helix (TMH) X of subunit I. This conformationally-dynamic section has been suggested to link the redox activity with the putative H pathway of proton transfer in mammalian C*c*O. The two helix X mutants, Val380Met (V380M) and Gly384Asp (G384D), generated in the genetically-tractable yeast C*c*O, resulted in a respiratory-deficient phenotype caused by the inhibition of intra-protein electron transfer and C*c*O turnover. Molecular aspects of these variants were studied by long timescale atomistic molecular dynamics simulations performed on wild-type and mutant bovine and yeast C*c*Os. We identified redox- and mutation-state dependent conformational changes in this span of TMH X of bovine and yeast C*c*Os which strongly suggests that this dynamic module plays a key role in optimizing intra-protein electron transfers.

## Introduction

**FIGURE 1:**
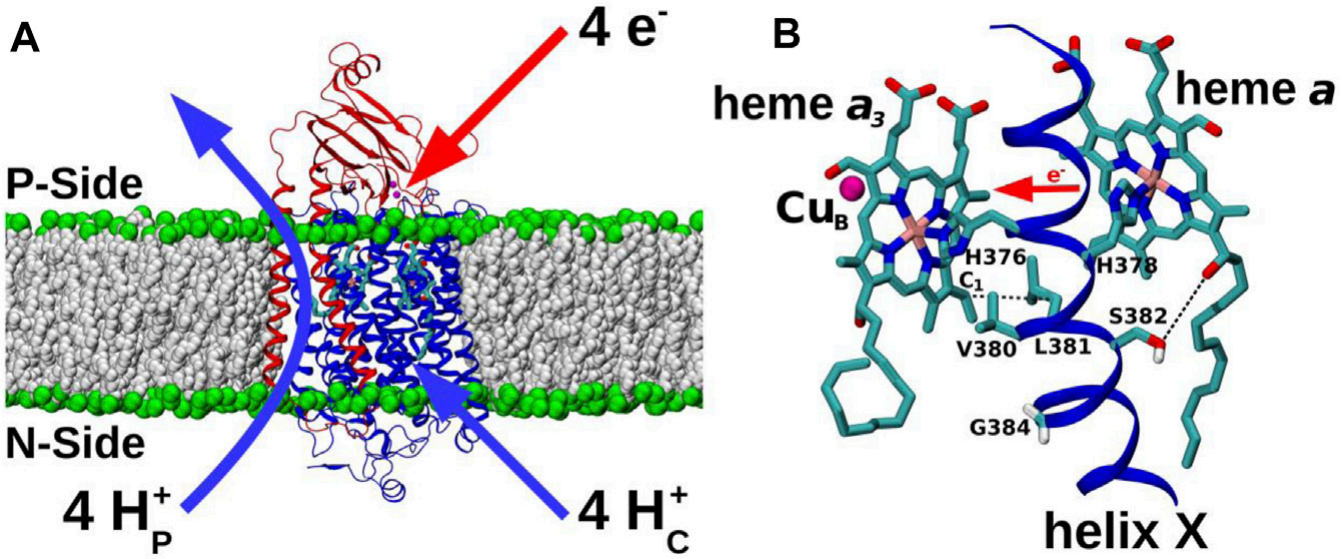
C*c*O and TMH X of catalytic subunit I. **(A)** Subunit I (blue) and II (red) of C*c*O from yeast *Saccharomyces cerevisiae* (PDB 6HU9) embedded in a POPC lipid bilayer (grey and green spheres). Redox-active centers are included, and the overall proton transfers (H^+^_P_ – pumped protons; H^+^_C_ – substrate protons) associated with a full four-electron catalytic cycle are indicated. P-side: positively-charged intermembrane space; N-side: negatively-charged mitochondrial matrix. **(B)** Redox-active cofactors of yeast C*c*O, heme *a*_3_ and heme *a* and their ligands H376 and H378, respectively, from TMH X of subunit I are shown. Cu_B_ (magenta sphere) and heme *a*_3_ form the BNC. Residues V380, L381, S382, and G384 are labelled. S382 sidechain oxygen – heme *a* hydroxyl oxygen and L381 C_β_ – heme *a*_3_ vinyl C_1_ distances are indicated by dashed black lines. Forward direction of heme-heme electron transfer is marked with a red horizontal arrow.

All forms of cytochrome *c* oxidase (C*c*O) reduce molecular oxygen (O_2_) to water with four electrons provided by cytochrome *c* and four protons from the mitochondrial matrix or bacterial cytoplasmic aqueous phase. These charge transfers from the two opposite sides of the membrane result in membrane polarization, which is further enhanced with an energetically uphill transfer of four more protons across the membrane (Wikström, 1977) (Figure 1A). The resulting proton concentration difference and charge imbalance across the membrane (the protonmotive force) drives the synthesis of ATP. The reduction of O_2_ occurs at a highly conserved binuclear center (BNC) comprising heme *a*_3_ and Cu_B_. The redox chemistry at the BNC drives the proton pump of C*c*O. Extensive structural (Iwata et al., 1995; Svensson-Ek et al., 2002; Tsukihara et al., 1995; Abramson et al., 2000), biochemical (Pfitzner et al., 2000; Fetter et al., 1995; Maréchal et al., 2012) and computational work (Sharma et al., 2013; Sharma et al., 2015; Kaila et al., 2008; Ghosh et al., 2009; Popović and Stuchebrukhov, 2004) on C*c*Os from varying sources has delineated a now widely-accepted mechanism of proton pumping in bacterial and yeast enzymes, based on a D channel role for redox-coupled proton movements (Rich, 2017; Wikström and Sharma, 2018). However, high-resolution structural data on C*c*O from bovine heart mitochondria (Yoshikawa and Shimada, 2015), has led to a coupling mechanism proposal which instead involves the transport of the additional pumped protons *via* an alternative H pathway. Mutations in this H pathway in bacterial and yeast enzymes have not affected catalytic turnover or proton pumping (Lee et al., 2000; Maréchal et al., 2020), prompting the suggestion that their H pathways may instead have a dielectric role that could aid internal electron transfer or mediate allosteric effects (Rich and Maréchal, 2013; Sharma et al., 2017; Malkamäki et al., 2019). This dichotomy of possible proton pumping mechanisms between bacterial/yeast and mammalian C*c*Os remains an unresolved issue, hindered in particular by technical difficulties in introducing equivalent point mutations into the mammalian mitochondrial DNA-encoded subunits of respiratory complexes [cf. (Mok et al., 2020)]. This leaves computational approaches, as applied here, as ideal methods to explore function, to predict effects of such point mutations on enzyme mechanism and to compare them where possible with experimentally-verifiable effects of these mutations in genetically-viable organisms such as yeast. Here, we performed microseconds long atomistic molecular dynamics (MD) simulations on a high-resolution (1.5 Å) structure of oxidized bovine C*c*O (Yano et al., 2016), focusing on the highly conserved H376-G384 span of transmembrane helix (TMH) X of subunit I. The H378 and H376 are ligands of hemes *a* and *a*_3_, respectively, and the V380-G384 segment is a conformationally-flexible region (Figure 1B) that has been proposed to gate H channel proton conductivity *via* redox- and ligand-induced movement of S382 (Yoshikawa and Shimada, 2015). The MD simulations were extended with additional computations in both bovine and yeast C*c*Os, together with functional studies in yeast C*c*O, of two point mutations, Val380Met (V380M) and Gly384Asp (G384D). These two mutations were initially identified in random mutagenesis trials (Ortwein et al., 1997; Meunier and Rich, 1998) and cause loss of C*c*O turnover activity, thus highlighting the importance of this region of helix X in the enzyme mechanism. The simulations predict redox- and mutation-induced conformational transitions in yeast and bovine C*c*Os, based on which we suggest that this dynamic and functionally critical module performs a similar function in both enzymes, perhaps controlling intra-protein electron transfer rather than having a role in proton transfers.

## Results

### Catalytic Activity and Redox Properties of WT and Mutant Yeast C*c*O

**FIGURE 2:**
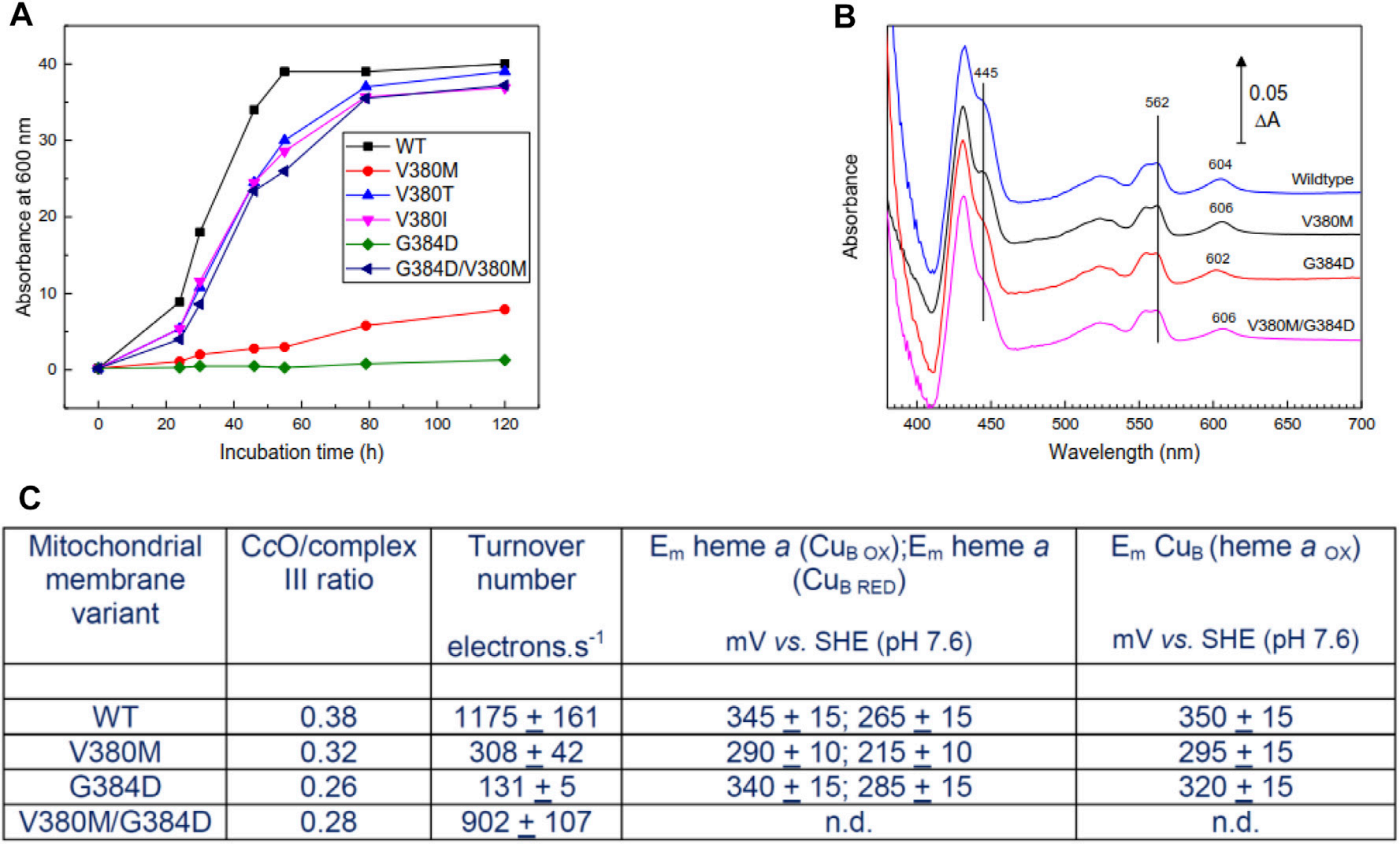
Properties of mutant yeast cells and mitochondrial membranes. **(A)** Respiratory growth rates of intact yeast cells in ethanol—containing medium. **(B)** Dithionite-reduced *minus* oxidized spectra of mitochondrial membranes derived from WT and mutant cell lines. Spectra were normalized to the same complex III concentrations assessed from ΔA at 562–575 nm. The 602–606 nm band arises primarily from heme *a*; hemes *a* and *a*_3_ contribute roughly equally to the 445 nm band. **(C)** Summary of ratio of C*c*O/complex III, C*c*O turnover numbers and redox properties of metal centers of C*c*O in mitochondrial membranes. Specific experimental details for all panels can be found in Methods. n.d. – not determined, but expected to resemble WT.

The two conserved residues V380 and G384 are located in the vicinity of S382, the proposed H channel gate in bovine C*c*O. Although yeast mutation S382A itself had no significant effect on respiratory competence, catalytic turnover or proton coupling (Maréchal et al., 2020), the two mutations (V380M and G384D) resulted in a severely respiratory-deficient phenotype caused by an assembled but inactive C*c*O (Figure 2) [see also (Ortwein et al., 1997; Meunier and Rich, 1998)]. V380M and G384D are located on the helix X face opposite to S382 and extend towards the BNC (Figure 1B), and are quite separate from all three proposed proton channels (Supplementary Figure S1). Mutations of these two residues are thus not expected to kill enzymatic activity by directly influencing the proton transfer reactions. Instead, due to their proximity to hemes, inhibitory effects on redox reactions are possible. The inhibitory effects, together with the known conformational flexibility and very strong conservation of this span of TMH X (Supplementary Figure S2), certainly point to this domain having a crucial role to play.

Cell respiratory growth profiles (Figure 2A) confirmed the respiratory-deficiency phenotype of the V380M and G384D point mutations. The revertants M380T and M380I (V380T and V380I, respectively) restored respiratory competence as, surprisingly, did the double mutant V380M/G384D [Figure 2A, see also (Namslauer et al., 2011)]. The inhibition of growth of V380M and G384D cells was not due to impaired expression of C*c*O, which was expressed above WT levels (3.2 and 2.6, respectively, compared to 2.4 nmol/gm wet weight in WT). C*c*O contents and turnover numbers in mitochondrial membranes further confirmed that respiratory deficiency arose primarily from impairment of C*c*O turnover in the point mutants and its recovery in the double mutant (Figures 2B,C). Reduced *minus* oxidized difference spectra revealed a 2 nm blueshift in G384D and a 2 nm redshift of the heme *a* α-band in V380M which surprisingly persisted in the double mutant (Figure 2B). In contrast, the α-band of heme *a*_3_ was not noticeably shifted, based on the reduced+CO *minus* reduced difference spectra which arise solely from heme *a*_3_ (Supplementary Figure S3). The redox behavior of WT and mutant yeast C*c*O was studied in the cyanide-inhibited mitochondrial membranes (see methods and Supplementary Figure S4). A rough estimate of the redox properties of heme *a* in cyanide-inhibited membranes (Figure 2C) indicated a lowering of its redox potential in V380M but little or no change in G384D. In the cyanide-inhibited state heme *a*_3_ is fixed in its oxidized state, but Cu_B_ still changes redox state and distorts the redox curve of heme *a* through electrostatic interaction; hence, the detailed shape of the heme *a* reduction *versus* E_h_ plot also provides information of the midpoint potential of Cu_B_ (Moody and Rich, 1990). This analysis suggested a lowering of E_m_(Cu_B_) in V380M but a much smaller possible decrease in G384D. In WT yeast cells, heme *a* is typically around 5–10% reduced during steady state turnover. Heme *a* remained fully reduced during steady state aerobic turnover in whole G384D cells, showing definitively that the inhibited step was electron transfer into the BNC. This was less clear in V380M cells, where heme *a* remained 40–50% reduced during aerobic turnover. Overall, these spectroscopic and redox data indicate structural perturbation of heme *a* in both mutants. The reason for inhibition in G384D is most likely a low E_m_ of heme *a*_3_ which prevents electron transfer from heme *a*. The cause of inhibition of V380M is less clear, but most likely involves electron transfers both into and from heme *a*. Hence, both mutations V380M and G384D in the conserved segment V380-G384 perturb internal electron transfer reactions of C*c*O through redox and/or structural changes. The presence of both mutations together must alleviate the effects of the single mutations in order to restore turnover and respiratory competence.

### Molecular Dynamics Simulations of WT and Mutant Forms of Yeast and Bovine C*c*Os

**FIGURE 3:**
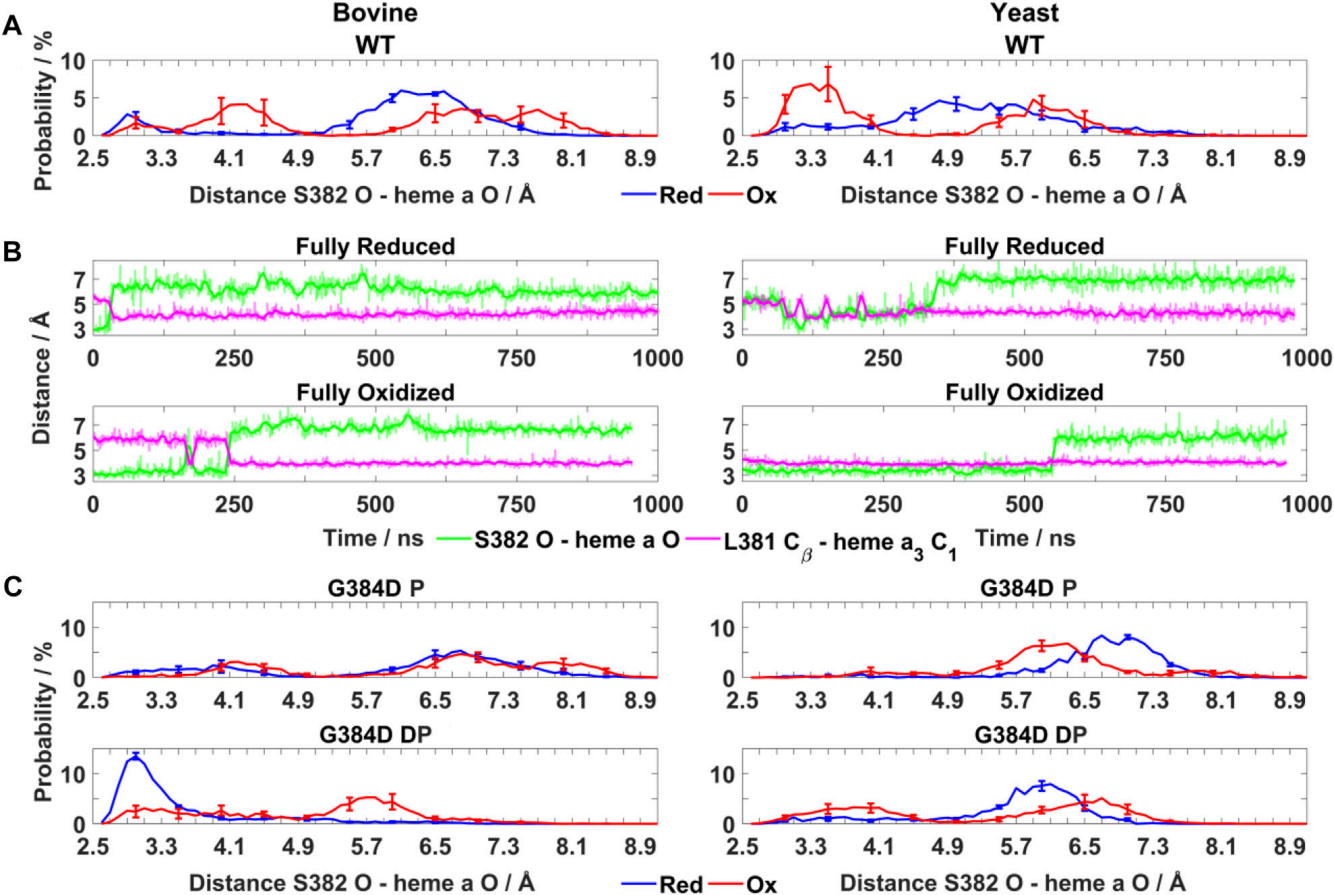
Conformational dynamics of TMH X residues in WT and mutant bovine and yeast C*c*Os. **(A)** Probability distribution (%) of S382 hydroxyl (O atom) – heme *a* farnesyl hydroxyl (O atom) distance in bovine and yeast WT C*c*O in the fully reduced and oxidized states. **(B)** Typical time-series of the S382 hydroxyl – heme *a* farnesyl hydroxyl and L381 C_β_ – heme *a*_3_ C_1_ distance in bovine **(left)** and yeast **(right)** WT C*c*O in the fully reduced and oxidized states. **(C)** Probability distribution (%) of S382 hydroxyl – heme *a* farnesyl hydroxyl distance in G384D bovine **(left)** and yeast **(right)** C*c*Os with D384 (P - protonated/DP - deprotonated) in their fully reduced and oxidized states. The distributions are averaged over all simulation replicas and error bars correspond to standard deviations.

To obtain molecular insights into the loss of oxidoreductase activity in the helix X mutants, we performed fully atomistic classical MD simulations of WT and mutant yeast and bovine C*c*Os in both oxidized and fully reduced states. Redox-dependent changes in distances between the S382 sidechain and heme *a* farnesyl hydroxyl, and between L381 (C_β_) and the C_1_ atom of the vinyl group of heme *a*_3_ (Figure 1B) were identified in high-resolution structural data of bovine C*c*O and their functional importance was emphasized (Shimada et al., 2017). In agreement with these structural data, in both bovine and yeast WT C*c*O simulations we observed a shorter S382 – farnesyl hydroxyl (heme *a*) distance (3–4 Å) in the fully oxidized state in comparison to the fully reduced state (greater than 4.5 Å) (Figure 3A). In further agreement are the coupled dynamics of the L381 (C_β_) – heme *a*_3_ vinyl (C_1_) distance which lengthens when the S382 – farnesyl hydroxyl distance shortens. This coupling was found to be more strongly-linked in bovine in comparison to yeast C*c*O simulations (Figure 3B). Overall, however, these simulated structural changes closely resemble the experimentally-observed conformational states and suggest that the *in-silico* model predictions are accurate.

**FIGURE 4:**
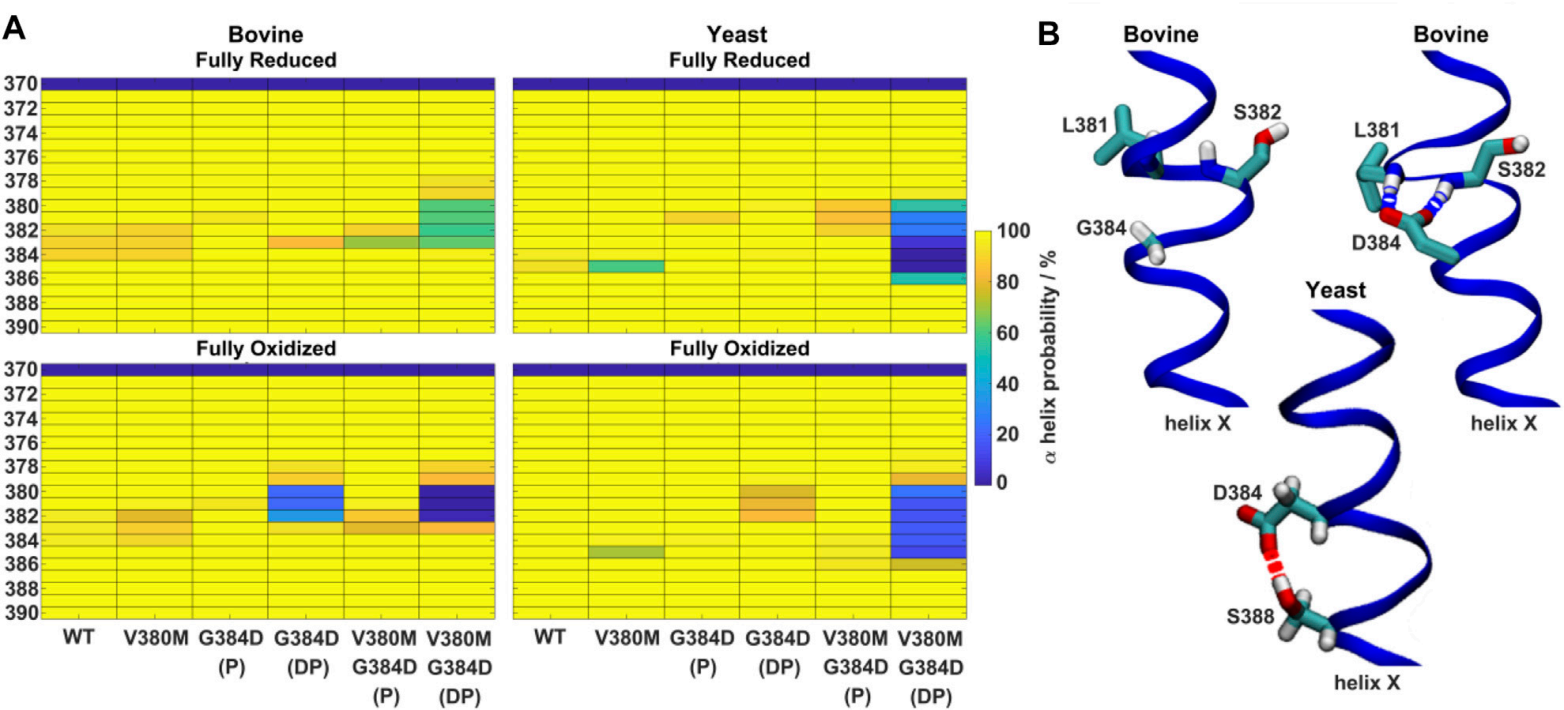
α-helicity of TMH X and hydrogen bonding rearrangements. **(A)** α-helix probability (%) of TMH X in WT, V380M, G384D (P/DP) and double mutant V380M/G384D (P/DP) bovine **(left)** and yeast **(right)** C*c*Os in their fully reduced **(top)** and oxidized **(bottom)** states. P (protonated) and DP (deprotonated) denote the protonation state of D384. **(B)** Conformation of TMH X in WT and the G384D (DP) mutant of bovine C*c*O **(top)** and the G384D (DP) mutant of yeast C*c*O **(bottom)**. L381, S382, G384/D384, and S388 are labelled. Hydrogen bonds connecting D384 with the backbones of L381 and S382 or the sidechain of S388 are indicated by dashed blue and red lines, respectively.

Next, the behaviors of bovine and yeast C*c*Os were simulated with the G384D mutation in both anionic and neutral forms (DP and P forms, respectively, see Supplementary Table S1). While neutral D384 (P) didn’t perturb much the geometry of TMH X in bovine or yeast C*c*O simulations, its anionic D384 (DP) form caused a large structural change resulting in significant loss of α-helicity in the 380-384 region, primarily in their fully oxidized states (Figure 4A). In contrast, in the fully reduced states, α-helicity was maintained and was similar to WT. Due to the electrostatic repulsion from reduced hemes, anionic D384 (DP) was displaced away from hemes by ca. 1–2 Å (D384 Cα – heme *a*_3_ Fe distance) from its original position, and formed hydrogen bonds to water molecules which diffused into the protein interior (ca. 7.4 ± 1.6 and 8.0 ± 1.8 water molecules with D384 locus) for ca. 95.6 ± 1.1% and 83.4 ± 1.1% of bovine and yeast C*c*O simulation times, respectively. On the other hand, in the fully oxidized state, anionic D384 (DP) was stabilized with strong hydrogen bonds to backbones of L381 and S382 (70.3 ± 7.3%) in bovine C*c*O and the sidechain of unique S388 (61.2 ± 3.4%) in yeast C*c*O (Figure 4B), resulting in a dominant loss of helicity. Overall, with neutral D384 (P), the TMH X helicity was preserved as in WT enzyme for both redox states in both enzymes. However, with anionic D384 (DP), a loss of helicity was observed in the oxidized state for both yeast and bovine C*c*Os. This suggests that it is most likely the anionic nature of aspartate in oxidized state of enzyme that perturbs the WT conformation of TMH X and is in part the reason for the loss of catalytic activity of yeast C*c*O.

As noted above, the S382 – farnesyl hydroxyl (heme *a*) distance displays redox-state dependency (Figure 3A), we therefore next studied how this distance behaved in the G384D mutant. The bovine G384D (DP) simulation data showed stabilization of a shorter S382 – farnesyl hydroxyl distance in the fully reduced state of enzyme, in stark contrast to WT simulations (Figures 3A,C). A similar effect was less clear in simulations of the lower-resolution yeast structure. However, in both yeast and bovine C*c*O simulations, the S382 – farnesyl hydroxyl distance, which was found to be 3–4 Å in the WT fully oxidized state (Figure 3A), was found to partly lengthen in the G384D (DP) case (Figure 3C). This was also observed when bovine and yeast C*c*O simulations were performed with neutral G384D (P) (Figure 3C), suggesting that the G384D mutant disrupts the functionally important redox-state dependency of the S382 – farnesyl hydroxyl (heme *a*) distance.

Overall, the above data highlight that the loss of α-helicity, redox-state dependent hydrogen bonding re-arrangements as well as perturbation of redox-state dependent conformational dynamics of the 380-384 region of helix X may all contribute to the observed loss of activity in the yeast G384D C*c*O mutant. Given that both bovine and yeast C*c*O share high structural and sequence similarity in the TMH X region (Supplementary Figure S2), and given the resemblance of the conformational transitions of S382 – farnesyl hydroxyl distance and TMH X helical propensity in WT and mutant enzymes, it is expected that a similar activity loss would be found in an equivalent mutant of bovine C*c*O, which currently remains inaccessible to genetic manipulation.

In addition to these conformational effects in the G384D mutant, our density functional theory (DFT) calculations on small and large model systems (see Computational Methods) revealed a lower electron affinity of heme *a*_3_ in the anionic G384D (DP) mutant compared to the WT enzyme (by ca. −11.5 to −9.1 kcal/mol, Table 1). In contrast, when modeled with neutral G384D (P), WT like electron affinity was observed. The effect of lowered electron affinity was also seen when DFT calculations were performed on WT and anionic G384D (DP) MD simulation snapshots (*n* = 5, see methods). We suggest that in addition to the conformational effects discussed above for G384D mutant, lowered electron affinity of heme *a*_3_ also contributes to the observed enzymatic inactivity, and is in agreement with our experimental data that show that electron transfer from heme *a* to heme *a*_3_/Cu_B_ is blocked.

**TABLE 1:** Electron affinity of heme *a*_3_ in WT and mutants.

System		∆(OX-RED) - ∆(OX-RED)_WT_ kcal mol^−1^
(heme *a*_3_) WT		0.0
(heme *a*_3_) G384D	Protonated D384	1.8
	Deprotonated D384	−11.4 ± 0.1^d^ (−9.1)^a^
	Deprotonated D384 + Water^b^	−12.4
(heme *a*_3_) V380M	Short (M380-Fe distance)	−2.5 (-2.3)^a^
	Long (M380-Fe distance)	0.7
	Weighted^c^	−1.2
(heme *a*_3_) V380M/G384D	Protonated D384	0.2
	Deprotonated D384	−10.2
	Deprotonated 384 + Water^b^	−14.3

a Large model system (see computational methods).

b Water molecules within 6 Å of residue 380 or 384.

c Weighted according to the short/long distribution in corresponding MD simulations.

d Mean and standard deviation is based on six independent DFT calculations (see methods).

**FIGURE 5:**
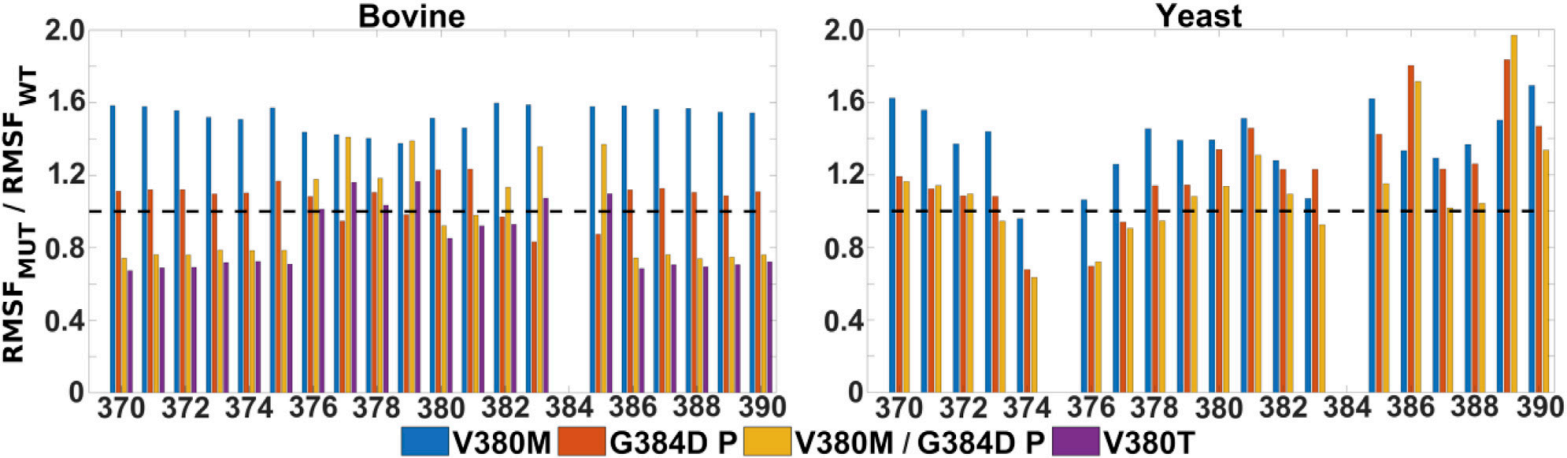
Mobility of TMH X residues in WT, single and double mutant bovine and yeast C*c*O simulations. Ratio of RMSF (root mean square fluctuations) of the sidechains of helix X from mutant and WT simulations. Bovine **(left)** and yeast **(right)** C*c*O simulations in their fully oxidized states are displayed with data from V380M (blue), protonated G384D (P, red), protonated V380M/G384D (P, yellow) and V380T (purple). The dashed black line (y = 1) indicates a WT-like RMSF.

**FIGURE 6:**
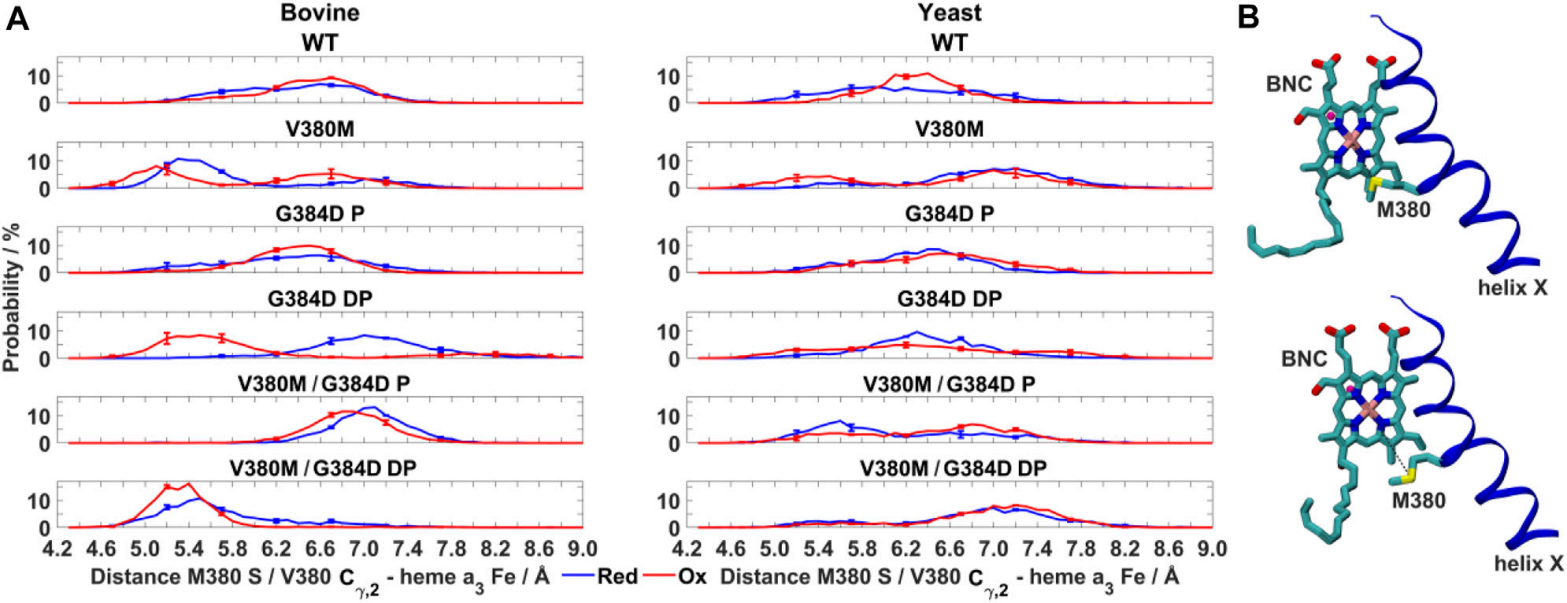
Dynamics of V380M mutant in bovine and yeast C*c*O. **(A)** Probability distribution (%) of V380/M380– heme *a*_3_ Fe distance in WT, V380M, and V380M/G384D (P/DP) bovine **(left)** and yeast **(right)** C*c*Os in their fully reduced and oxidized states. **(B)** Conformation of the BNC and TMH X in bovine V380M C*c*O simulations. Residue M380 is labelled. The M380 S – heme *a*_*3*_ Fe distance (short, top and long, bottom) is indicated by dashed black lines. The distributions are averaged over all simulation replicas and error bars correspond to standard deviations.

In contrast to the blocked electron transfer from heme *a* to heme *a*_3_ observed in the G384D mutant, the reduction of heme *a* was most likely slowed in the V380M mutant due to the perturbation in its environment and the drop in its midpoint potential (Figure 2). In the V380M mutant simulations, we found a partial loss of helicity in TMH X (Figure 4A) and an increased sidechain fluctuation along the helix (Figure 5). These effects were seen in both bovine and yeast C*c*Os. In addition, we observed two distinct populations of the methionine sidechain in both yeast and bovine C*c*O MD runs (Figures 6A,B), while no such effect was observed with V380 in WT and G384D mutant simulations. The M380 conformation vicinal to heme *a*_3_ (5–5.5 Å) was found to induce a weak effect of lowered electron affinity of heme *a*_3_ (−2.50 to −2.33 kcal/mol in small and large DFT cluster models, Table 1) relative to the WT valine. Overall, these data point to electron transfer into heme *a* and onwards being likely hindered in the V380M mutant by perturbation in electron affinities, primarily of heme *a* and possibly Cu_B_, resulting from the structural changes induced in helix X residues.

It is remarkable that the yeast C*c*O double mutant carrying both V380M and G384D mutations is fully respiratory competent with close to WT C*c*O turnover activity (Figure 2). As stated above, the structural and redox changes caused by the single mutations must be ameliorated when both mutations are present together. In our simulations of fully oxidized bovine C*c*O, the double mutant with anionic G384D (DP) showed a loss of α-helicity similar to that found in the single anionic G384D (DP) mutant (Figure 4A). Similar to bovine C*c*O, the yeast C*c*O double mutant with anionic G384D (DP) also showed α-helicity loss in helix X in both redox states. In contrast, in both bovine and yeast C*c*Os with neutral G384D (P), α-helicity of helix X remained similar to WT in both single and double mutants and in both redox states (Figure 4A). Likewise, sidechain fluctuations along the TMH X with neutral G384D (P) in both single and double mutants are rather comparable to WT, and also to the respiratory competent yeast mutant V380T when simulated in oxidized bovine C*c*O (Figure 5). This raises the possibility that the restored activity of the double mutant might be because the neutral state of D384 is preferred, hence preventing significant loss of helicity of helix X and, hence, minimizing redox potential changes. Indeed, MD simulation data reveal that there are fewer water molecules in the vicinity of anionic D384 (DP) in the double mutant (3.9 ± 2.9) compared to the single mutant G384D (7.3 ± 1.5), further suggesting that lower level of hydration may stabilize the neutral state of G384D (P) in the double mutant case. When we analyzed the dynamics of M380, which showed two distinct populations (vicinal and distal to heme *a*_3_ configurations, Figure 6), we found the vicinal conformation was entirely lost in the neutral D384 (P) double mutant, and the distal configuration retained higher occupancy in bovine simulations. Thus, we propose that the latter conformation, as well as the neutral state of D384 (P), would render the electron affinities of the hemes close to WT values, hence sustaining normal oxidoreductase activities. This is also supported by our DFT calculations which show a WT-like electron affinity of heme *a*_3_ in the double mutant (Table 1), suggesting that D384 may indeed attain a neutral state thus rescuing the respiratory activity.

## Discussion

To date the highest resolution structures of *Bos taurus* C*c*O (1.5 Å) were obtained by X-ray crystallography. These structures have provided remarkable insights into its protein interior (Yano et al., 2016). However, due to current limitations on mammalian mitochondrial genome editing, mutation-based experimental tests of predicted functions using single point mutations have not been available to confirm whether a unified underlying mechanism operates in between C*c*Os from different domains of life. Yeast mitochondrial C*c*O, a near evolutionary relative of mammalian C*c*Os, was genetically altered to study effects of two point mutations (V380M and G384D) in the conserved H376-G384 span of TMH X, combined with molecular dynamics simulations of WT and mutant enzymes. From WT C*c*O simulations, we confirmed the known concerted redox-coupled dynamics of the S382 sidechain - heme *a* farnesyl hydroxyl distance with that of the L381 (Cβ) - C_1_ of vinyl group of heme *a*_3_ distance. This provided confidence into the modeling and simulation protocol applied in this study. The yeast G384D mutation caused a blockage of electron transfer from heme *a* to the BNC most likely due to the observed MD-based conformational and hydrogen-bonding changes in the TMH X segment of both bovine and yeast C*c*O, together with the negative charge of aspartate, causing a lowered midpoint potential of heme *a*_3_, a notion supported by DFT calculations. Simulations of the V380M mutant of both bovine and yeast C*c*Os showed a higher level of flexibility in helix X residues as well as stabilization of a unique conformation of methionine closer to heme *a*_3_. These lead to redox potential changes of heme *a*, and also possibly of the binuclear center, hence inducing the decrease in enzymatic activity. The restored activity of double mutant V380M/G384D is indeed remarkable and puzzling at the same time. Our combined experimental and simulation data provide indirect support for the neutral state of aspartic acid in the V380M/G384D double mutant, in contrast to its more favored anionic character in the single mutant G384D. However, further direct investigations of protonation state, for example by FTIR spectroscopy, would be needed to confirm this proposal. Overall, comparisons of our MD simulations of mutant yeast and bovine C*c*Os in two different redox states reveal several similarities (as detailed in Results section), but also some differences, which in part are likely to arise from the lower resolution of the yeast C*c*O structure. Nevertheless, given high sequence similarity and the similarity of simulated behaviors of this TMH X span, we envisage that it fulfills the same functions in both bovine and yeast C*c*Os, and that introduction of the same mutations into bovine C*c*O would result in the same loss of function.

Our integrated approach combining yeast biochemistry and molecular simulations reveals that perturbation of the conformation of conserved helix X segment (residues 380–384) can have a profound effect on internal electron transfer reactions of C*c*O. This is in line with the observations from structural analysis, where this segment undergoes conformational changes depending on the redox state of the enzyme (Yoshikawa and Shimada, 2015). However, since electron transfer from heme *a* into the BNC is obligatorily coupled to proton transfer into the proton trap/loading site (Rich, 2017; Wikström and Sharma, 2018), turnover inhibition could also arise from interference in this linked proton transfer. We note that these helix X mutations are 10–15 Å from the three putative proton channels, but are much closer to the redox-active hemes *a* and *a*_3_ (Figure 1 and Supplementary Figure S1), which suggests a more direct effect of mutations on redox transitions than proton transfers in the three channels. Moreover, the distance of helix X residues from E242 of the D channel is 13–15 Å and from the proton trap is ca. 13 Å. Hence, assuming the widely held model of proton translocation *via* these elements (Rich, 2017; Wikström and Sharma, 2018), it is unlikely to have any major influence on proton movements *via* this route. The mutations are however closer to the S382 residue (ca. 6 Å), which resides on the opposite side of TMH X and is a key element of the H channel proton pump proposal (Yoshikawa and Shimada, 2015). However, H^+^/e^−^ coupling data from yeast C*c*O show no effect upon mutation of S382 to alanine (Maréchal et al., 2020), which argues against a role in H channel proton transfers. It is well-known that heme-heme electron transfer rate is extremely fast (ns tunneling) in C*c*O due to the proximity of the two heme edges (Verkhovsky et al., 2001). This fast electron transfer is unlikely to be sufficiently slowed by the point mutations discussed above to cause overall inhibition. Instead, we suggest that it is the overall slowing of the net electron transfer rate caused by changes in the redox potentials of cofactors that results in the mutant-induced turnover inhibition. Thus, we propose that the natural conformational dynamics of this critical segment may well control and facilitate intra-protein electron transfer (between heme *a* and the BNC), rather than having primary role in facilitating and gating a proton pumping channel.

## Experimental Methods

### Yeast Mutant Constructs and Mitochondrial Membrane Preparation

Yeast extract was purchased from Ohly GmbH, Germany. All other reagents were purchased from Sigma Aldrich. Yeast *Saccharomyces cerevisiae* strains were constructed from a modified strain W303-1B (Alpha *ade2 HIS3 leu2 trp1 ura3*) that expressed wild type C*c*O with a 6-his tag sequence attached to *Cox13* for ease of C*c*O purification**.** The respiratory growth defective mutants V380M and G384D were obtained by random mutagenesis as described in (Meunier et al., 1993) [see also (Ortwein et al., 1997; Meunier and Rich, 1998)]. The respiratory growth competent mutants or revertants V380T/I and V380M/G384D were derived from V380M and G384D by direct selection on respiratory medium. Subsequent confirmatory sequencing revealed that V380M and its revertants also had a silent mutation A308T. Protocols for growth of the yeast cells in galactose-containing medium and preparation of mitochondrial membrane fragments were as detailed in (Meunier et al., 2012). Mitochondrial membranes were either assayed immediately after preparation or were stored at −80°C in 50 mM potassium phosphate, 2 mM potassium EDTA at pH 7.4.

### C*c*O Content and Steady State Redox Poise in Whole Cells

For quantitation of level of expression of C*c*O, cells were resuspended to 80 mg wet weight cells/mL in 440 mM sucrose (to prevent sedimentation) and 50 mM potassium phosphate at pH 7.2. They were left for 1 min before scanning a baseline. 10 µM myxothiazol and 0.01% w/v hydrogen peroxide were then added to ensure that CcO was fully oxidized and a new spectrum was recorded. Dithionite was then added to fully reduce components and a further spectrum was taken after stabilisation. CcO was quantitated at 604–619 nm (WT), 606–621 nm (V380M and V380M/G384D) and 602–617 nm (G384D) using an extinction coefficient of 26 mM^−1^ cm^−1^ (Rich and Moody, 1997).

In order to determine the steady state redox poise of heme *a* and cytochrome *c*, cells were diluted to 20 mg/ml into aerobic 50 mM potassium phosphate (pH 7.2) and 440 mM sucrose. A baseline spectrum was recorded rapidly before anaerobiosis occurred. After anaerobiosis, causing C*c*O and cytochrome *c* to become fully reduced, a second scan was taken. Finally, 10 µM antimycin A (or 10 µM myxothiazol) and 0.01% w/v (∼3 mM) H_2_O_2_ were added and the sample was rescanned. These rapidly blocked the respiratory chain and released oxygen into the medium, causing C*c*O and cytochrome *c* to become fully oxidized. From these spectra, the total C*c*O concentration and the aerobic steady state redox poise of heme *a* and cytochrome *c* could be deduced.

### Turnover Numbers in Mitochondrial Membranes

C*c*O concentrations were measured from sodium dithionite-reduced *minus* oxidized difference spectra of mitochondrial membranes resuspended in 50 mM potassium phosphate and 2 mM EDTA pH 7.4. CcO was quantitated at 604–619 nm (WT), 602–617 nm (G384D) or 606–621 nm (V380M and V380M/G384D) with an extinction coefficient, Δε, of 26 mM^−1^ cm^−1^ (based on Δε at 606–621 nm of bovine C*c*O (Rich and Moody, 1997). Steady-state oxygen consumption rates were measured in a stirred reaction vessel of a Clark-type O_2_ electrode at 25°C. Assays were carried out using mitochondrial membranes containing 2–10 nM C*c*O in 10 mM potassium phosphate at pH 6.6, 50 mM KCl, 0.05% (w/v) UDM, 50 µM horse heart cytochrome *c* and 40 µM TMPD (Dodia et al., 2014). A baseline was measured in the absence of cyt *c* and the reaction was initiated by addition of 2 mM sodium ascorbate. Turnover numbers (TN) are expressed in terms of the number of electrons transferred from cyt *c* per second per C*c*O (e.s^−1^).

### Redox Potential Determinations

Redox behaviour of the 602–6 nm bands were determined in cyanide-inhibited mitochondrial membrane fragments. Since cyanide binds to heme *a*_3_ and substantially lowers its midpoint potential, these titrations represent the redox behaviour of heme *a* only. However, Cu_B_ remains still redox-active and interacts electrostatically with heme *a*, splitting the heme *a* redox plot into high potential (Cu_B_ oxidized) and low potential (Cu_B_ reduced) waves (Moody and Rich, 1990). Mitochondrial membranes were suspended in a buffer of 50 mM potassium phosphate and 2 mM EDTA at pH 7.4 and 23°C. 2 µM horse heart cytochrome *c* and 40 µM potassium ferricyanide were then added to fully oxidize the CcO and a baseline spectrum from 500 to 650 nm was recorded. 5mM potassium cyanide was then added. This rapidly bound to heme *a*_3_, after which time the cytochrome *c* and heme *a* began to reduce slowly with a very slow leak of endogenous reductant that forms in all types of mitochondrial membrane preparations (probably arising from slow lipid and protein oxidations), allowing a series of spectra to be recorded as these components became reduced. Finally, full reduction of cytochrome *c* and heme *a* was induced by addition of 4 mM sodium ascorbate. At each fractional reduction step the ambient potential, E_h_, was calculated from the fractional reduction of cytochrome *c* at 550–542 nm using a midpoint value of +255 mV *vs.* SHE. Fractional reduction of the 602–6 nm band was determined from the size of the 602–6 nm peak relative to the weighted average of reference points either side of the peak at λ_max_ ± 16 nm. A correction was made for the small contribution of cytochrome *c* at these wavelength triplets by subtraction of the appropriate fraction of its 550–542 nm absorbance change (0.014, 0.013 or 0.0115 for the 602, 604 and 606 nm triplets, respectively). Data were fitted to a model in which heme *a* interacts anticooperatively with Cu_B_, resulting in high potential (Cu_B_ oxidized) and low potential (Cu_B_ reduced) components of heme *a* (Moody and Rich, 1990). Heme *a*_3_ redox potential determinations require accurate titration and deconvolution of the 445 nm band contributions of hemes *a* and *a*_3_ in the unligated enzyme. In contrast to bovine mitochondria, this cannot be achieved with sufficient accuracy in these yeast mitochondrial membranes due to their much lower C*c*O/complex III ratios.

## Computational Methods

### Molecular Dynamics Simulations

We performed molecular dynamics (MD) simulations of small model systems (∼100,000 atoms), representing the core subunits (SU) I and II of bovine and yeast C*c*O using a high resolution (1.5 Å) bovine [5B1A (Yano et al., 2016)] and a lower resolution (3.35 Å) yeast [*S. cerevisiae;* 6HU9 (Hartley et al., 2019)] structure.

To represent the C*c*O variants generated by site-directed mutagenesis, V380 and G384 of SU I were altered to M/T and D, respectively (Supplementary Table S1). The C*c*O model system was embedded in a bilayer of POPC lipids (85 lipids in the lower and upper leaflet) and solvated in TIP3P water with 0.15 M NaCl using CHARMM-GUI and associated tools (Jo et al., 2008). The redox-active sites were modeled in their fully oxidized state with H_2_O and OH^−^ as ligands of heme *a*_3_ and Cu_B_, respectively, and the catalytic tyrosine (Y244) in its anionic form or, in their fully reduced state, without the oxygenous ligands and Y244 in its neutral form (Johansson et al., 2008; Sharma et al., 2013). Residues E242, K319, and D364 of SU I were patched neutral while standard protonation states were assumed for all other residues except the mutated residue D384 which was modeled in both its neutral and anionic form. N- and C-terminals of the protein were treated by the CHARMM NTER and CTER patches, while the CHARMM force field was used for the protein (CHARMM22/36) (MacKerell et al., 1998; MacKerell et al., 2004; Best et al., 2012), membrane (CHARMM27/36) (Feller et al., 1997; Klauda et al., 2010), water (CHARMM36) (Jorgensen et al., 1983), and ions (CHARMM36) (Beglov and Roux, 1994).

All model systems were subjected to two consecutive energy minimizations using Gromacs 2019.3 (Abraham et al., 2015), with and without frozen protein, of 50,000 steps and a maximal force <1,000 kJ mol^−1^ nm^−1^ using the steepest decent algorithm and a subsequent equilibration of 0.2 ns using the Berendsen barostat (Berendsen et al., 1984), Nosé-Hoover thermostat (Nosé, 1984; Hoover, 1985), constraints on all bonds including hydrogen atoms [LINCS (Hess, 2008)], and a timestep of 2 fs. For the final production runs (see Supplementary Table S1 for individual simulation lengths) the Parrinello-Rahman barostat (Parrinello and Rahman, 1981) and Nosé-Hoover thermostat were applied. Analysis and visualization of all simulation trajectories was performed with VMD software (Humphrey et al., 1996) (Supplementary Figure S5).

To test the reversibility of the conformational changes of helix X, we performed additional WT simulations using the final protein conformations of the G384D and V380M/G384D mutant simulations with deprotonated D384 in the fully oxidized state of bovine C*c*O as starting point. These conformations were chosen because they showed the largest deviation of helix X from an α-helical structure. In all simulations an α-helical structure of helix X was restored when the mutations were reversed, thus further supporting our modeling and simulation protocol.

For the double mutant simulations, both V380 and G384 were mutated during the system setup. To complement these simulations, we performed six additional bovine double mutant simulations with anionic D384 in the fully oxidized state (Supplementary Table S1). Three of these simulations were started from simulations in which G384 was mutated to anionic D384 in the fully oxidized state. In two of these simulations anionic D384 formed (as before) stable hydrogen bonds to the backbones of L381 and S382, while no such hydrogen bonds were formed in the third simulation. The remaining three simulations were started from simulations in which V380 was mutated to M380 in the fully oxidized state (Supplementary Table S1). In two of these simulations anionic D384 formed (as before) stable hydrogen bonds to the backbones of L381 and S382, while the hydrogen bonds formed in the third simulation were rather unstable. Overall, similar results were obtained despite starting MD simulations from different initial conditions. This further strengthens data obtained from simulations and our results and conclusions.

p*K*a calculations were also performed on the bovine and yeast C*c*O MD simulation snapshots using Propka (Olsson et al., 2011; Søndergaard et al., 2011) and Delphi (Wang et al., 2015; Wang et al., 2016) software to estimate the p*K*a of D384 in single and double mutants and in different redox and protonation states. These calculations, however, did not support the neutral state of D384 in the double (V380M/G384D) mutant (see Results section).

### Density Functional Theory Calculations

We determined the energy difference between the oxidized and reduced state of heme *a*_3_ to investigate the role of the charge change of heme *a*_3_ in the different variants of C*c*O without the coupled proton transfer. The calculations were performed in two model systems (“small” and “large”), both constructed from the high-resolution bovine C*c*O structure (PDB 5B1A) and contained heme *a*_3_ in its reduced or oxidized state, as well as residues H376, V380 (or M380), and G384 (or D384) of SU I. The “large” model systems contain in addition residues F344, F348, L381, S382, and M383 of SU I. For M380 and D384 two states were considered, i.e., “vicinal”/“distal” (short/long) conformation of M380 according to MD simulations and protonated (P)/deprotonated (DP) form of D384, in the “small” model systems, while only the “vicinal” conformation of M380 and the deprotonated form of D384 was considered in the “large” model systems. To reduce the size of the model systems and to guarantee a charge close to neutral, the propionate groups of heme *a*_3_, the farnesyl chain of heme *a*_3_, and the backbones of all protein residues were replaced by fixed capping atoms (H). For “small” model systems with deprotonated D384, nine to twelve additional water molecules were included, according to the MD simulations. The remaining coordinates were obtained as before. In addition, we also performed DFT calculations on small model systems (*n* = 5) scooped out of the simulation snapshots obtained from WT and G384D (DP) bovine MD runs.

All model systems were subjected to a geometry optimization using TURBMOLE 7.3 (Ahlrichs et al., 1989) with the DFT functional BP86 (Perdew, 1986; Becke, 1988), a convergence criterion of 10^−6^ Hartree, and a Becke-Johnson damped dispersion correction (Grimme et al., 2010; Grimme et al., 2011). The Fe atom of heme *a*_3_ was treated by the def2-TZVP basis set, while all remaining atoms were treated by the def2-SVP basis set (Weigend and Ahlrichs, 2005). To speed up the geometry optimizations the multipole accelerated R-I approximation (Sierka et al., 2003) was used. Single-point energy calculations were performed for all optimized structures using the DFT functional B3-LYP (Lee et al., 1988; Becke, 1993) with the same convergence criterion and dispersion corrections as before. All atoms were treated by the def2-TZVP basis set. To account for the removed protein environment the conductor-like screening model (Klamt and Schüürmann, 1993) was used with a dielectric constant (*ε*) of four. Table 1 lists the data obtained from DFT calculations.

## Data Availability

All data are included in the article. The simulation snapshots are available upon request from the corresponding author VS, vivek.sharma@helsinki.fi.
