# Supplementary material for: Electron Transfer Coupled to Conformational Dynamics in Cell Respiration
Source: Front Mol Biosci. 2021 Aug 6;8:711436. doi: 10.3389/fmolb.2021.711436 (PMC8378252; doi:10.3389/fmolb.2021.711436)
Supplement: Supplementary file 1 [file DataSheet1.docx]

**Supplementary Information**

**Electron transfer coupled to conformational dynamics in cell respiration**

Marco Reidelbach^1^, Christoph Zimmer^2^, Brigitte Meunier^3^, Peter R. Rich^2,*^, Vivek Sharma^1,4*^

^1^ Department of Physics, PO Box 64, University of Helsinki, 00014 Helsinki, Finland

^2^ Department of Structural and Molecular Biology, University College London, Gower Street, London, WC1E 6BT, UK

^3^ Institute for Integrative Biology of the Cell (I2BC), Université Paris-Saclay, Gif-sur-Yvette, France

^4^ HiLIFE Institute of Biotechnology, PO Box 56, University of Helsinki, 00014, Helsinki, Finland

* Corresponding authors

Vivek Sharma, [vivek.sharma@helsinki.fi](mailto:vivek.sharma@helsinki.fi)

Peter Rich, [prr@ucl.ac.uk](mailto:prr@ucl.ac.uk)

**Table S1.** Bovine C*c*O (black font) and yeast C*c*O (blue font) model systems and simulation lengths. P (protonated) and DP (deprotonated) denote the protonation state of D384.

| **System** | **Fully Reduced** | | | **Fully Oxidized** | | |
| --- | --- | --- | --- | --- | --- | --- |
|  | **Run 1** | **Run 2** | **Run 3** | **Run 1** | **Run 2** | **Run 3** |
| WT | 1591 ns | 1043 ns | 879 ns | 954 ns | 1049 ns | 935 ns |
| V380M | 1361 ns | 984 ns | 1189 ns | 925 ns | 1042 ns | 1092 ns |
| G384D (P) | 1151 ns | 1024 ns | 1011 ns | 1033 ns | 1163 ns | 904 ns |
| G384D (DP) | 1213 ns | 987 ns | 1153 ns | 938 ns | 980 ns | 954 ns |
| V380M / G384D (P) | 875 ns | 871 ns | 909 ns | 1230 ns | 917 ns | 889 ns |
| V380M / G384D (DP) | 852 ns | 1056 ns | 1008 ns | 1029 ns | 1072 ns | 1038 ns |
| WT started from G384D (DP) | - | - | - | 497 ns | 449 ns | 501 ns |
| WT started from V380M/G384D (DP) | - | - | - | 453 ns | 442 ns | 493 ns |
| V380M / G384D (DP) started from V380M | - | - | - | 469 ns | 449 ns | 436 ns |
| V380M / G384D (DP) started from G384D (DP) | - | - | - | 475 ns | 490 ns | 502 ns |
| M380T | - | - | - | 495 ns | 487 ns | 478 ns |
| M380T (started from V380M) | - | - | - | 487 ns | 463 ns | 490 ns |
| WT | 1080 ns | 979 ns | 939 ns | 964 ns | 1178 ns | 910 ns |
| V380M | 1064 ns | 905 ns | 992 ns | 894 ns | 904 ns | 903 ns |
| G384D (P) | 1120 ns | 947 ns | 1023 ns | 843 ns | 921 ns | 999 ns |
| G384D (DP) | 1117 ns | 971 ns | 989 ns | 1065 ns | 917 ns | 927 ns |
| V380M / G384D (P) | 981 ns | 774 ns | 781 ns | 1230 ns | 758 ns | 837 ns |
| V380M/G384D (DP) | 973 ns | 775 ns | 775 ns | 1029 ns | 792 ns | 797 ns |

**Fig. S1. Transmembrane helix (TMH) X and proton channels.** Positions of V380 and G384 of helix X relative to possible proton channels in subunit I of yeast C*c*O are indicated. The 12-helical structure of yeast (*S. cerevisiae*) C*c*O (drawn using coordinates from PDB entry 6HU9) is viewed from the ‘top’, P-phase (intermembrane space) side. The TMH X (residues 369-397) backbone is shown in blue with residues V380, S382 and G384 highlighted. Principal residues that contribute to the three possible proton channels are highlighted in magenta (K channel), green (D channel) and yellow (H channel).

**
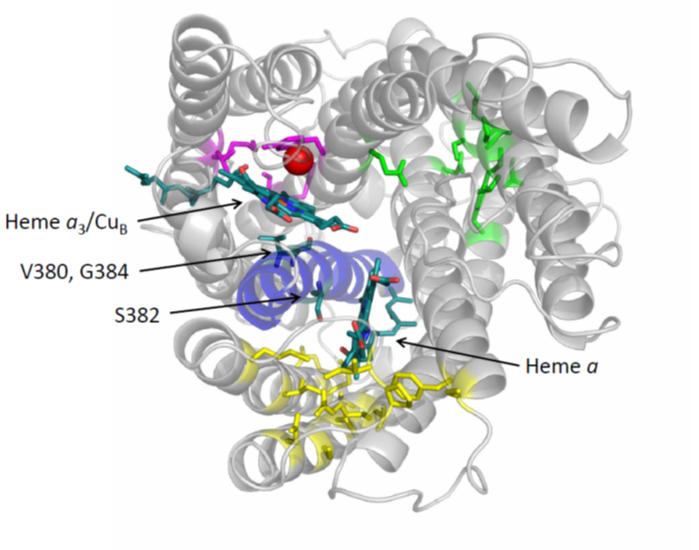
**

**Fig. S2. Conservancy of the TMH X segment in C*c*Os from different organisms.**

**
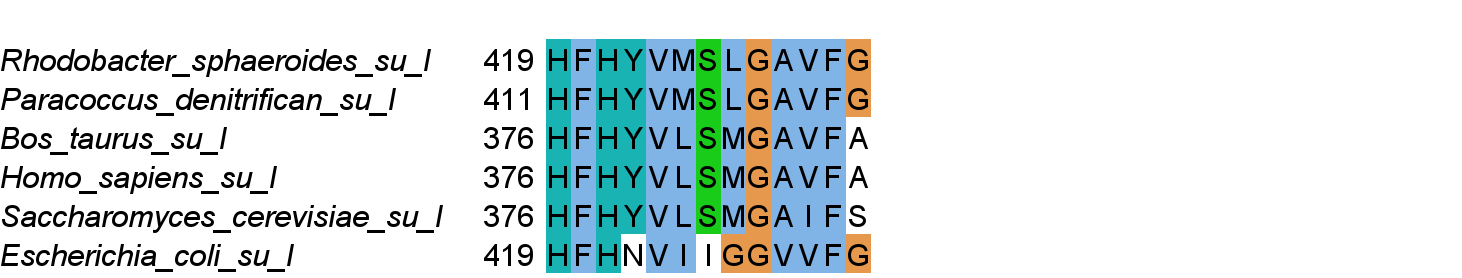
**

**Fig. S3. Carbon monoxide binding spectra of WT and mutant yeast mitochondrial membranes.** Mitochondrial membranes were diluted to approximately 0.18 µM C*c*O (based on reduced *minus* oxidized difference spectra) in 50mM potassium phosphate/2mM EDTA at pH 7.4. Sodium dithionite was added and, after full reduction had stabilized, reference spectra were recorded. The samples were then briefly bubbled with carbon monoxide and the reduced + CO *minus* reduced difference spectra shown were recorded.

**Fig. S4. Typical redox titration of the 602 nm band of cyanide-inhibited mitochondrial membrane fragments of the G384D mutant.** Titrations were performed as detailed in Methods. Membranes were suspended in a buffer of 50mM potassium phosphate and 2 mM EDTA at pH 7.4 and 23C. 2 µM horse heart cytochrome *c* and 40 µM potassium ferricyanide were then added to fully oxidize the C*c*O and a baseline spectrum from 500-650 nm was recorded. 5mM potassium cyanide was then added. This rapidly bound to heme *a*_3_, after which time the cytochrome *c* and heme *a* began to reduce slowly with a very slow leak of endogenous reductant, allowing a series of spectra to be recorded as these components became reduced. Finally, full reduction of cytochrome *c* and heme *a* was induced by addition of 4 mM sodium ascorbate. At each fractional reduction step the ambient potential, E_h_, was calculated from the fractional reduction of cytochrome *c* at 550-542 nm using a midpoint value of +255mV *versus* SHE. Fractional reduction of heme *a* was determined from the size of the 602 nm peak relative to the weighted average of reference points either side of the peak at λ_max_ + 16 nm. A correction was made for the small contribution of cytochrome *c* at these wavelength triplets. The fitted line is for a model in which heme *a* interacts anticooperatively with Cu_B_, resulting in high potential (Cu_B_ oxidized) and low potential (Cu_B_ reduced) midpoint potential components of heme *a* at 340 and 290 mV and a midpoint potential of Cu_B_ (with heme *a* oxidized) of 310 mV.

**Fig. S5. Structural stability in simulations.** Typical backbone (subunits I and II) RMSD (root mean square deviation) time-series in bovine (left) and yeast (right) C*c*Os. WT (blue), V380M (red), G384D P (green), G384D DP (magenta), V380M/G384D P (cyan) and V380M/G384D DP (yellow) C*c*O in the fully reduced (top) and oxidized (bottom) states. The flexible C terminus region (residues 496-514 and 480-534 in bovine and yeast C*c*Os, respectively) was removed from RMSD calculation. P – protonated, DP – deprotonated.

**
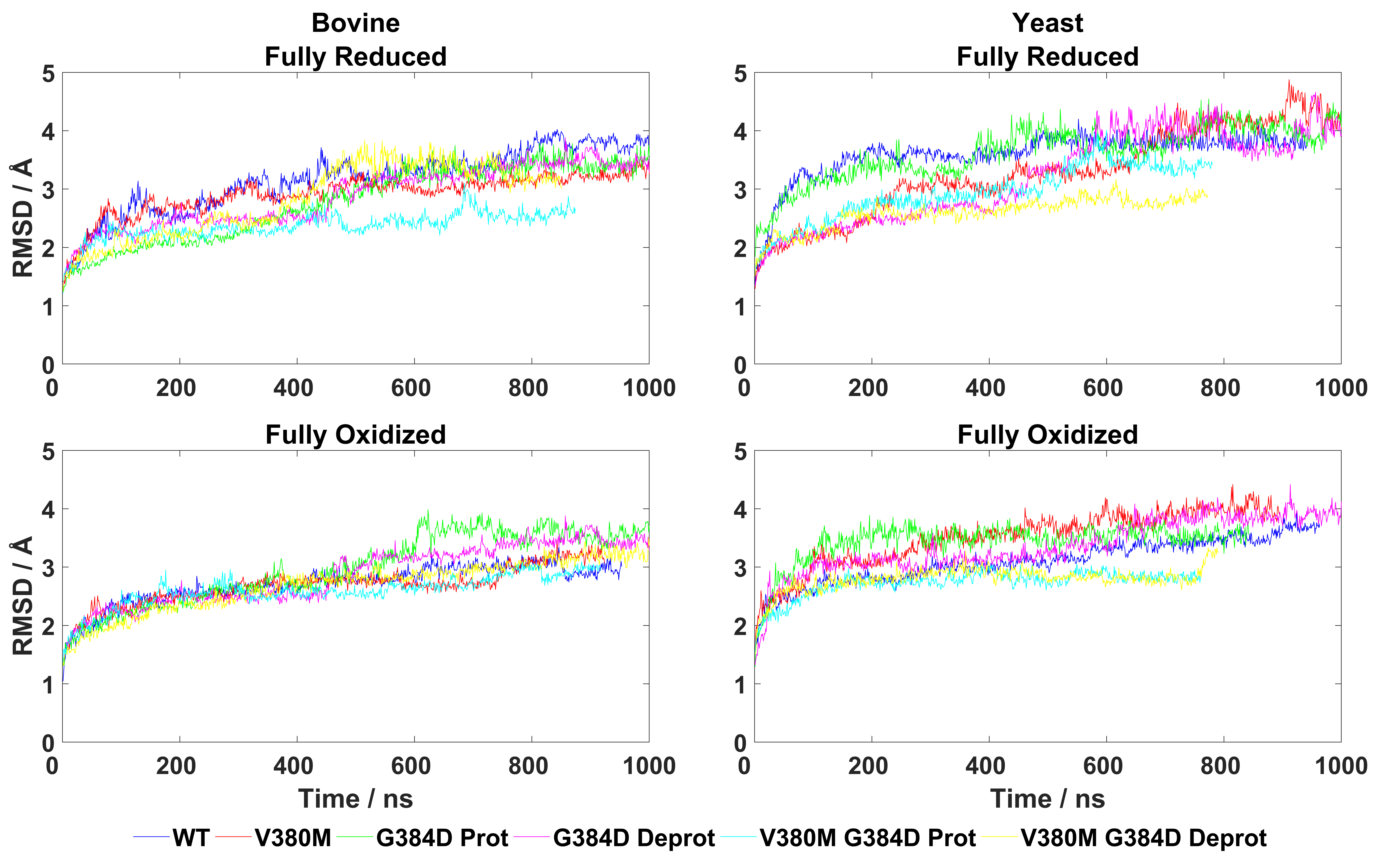
**
